# WIN-MTB-2024017 – WIN International Molecular Tumor Board: A 48-year-old female with multiple primary cancers

**DOI:** 10.18632/oncotarget.28899

**Published:** 2026-07-21

**Authors:** Wafik S. El-Deiry, Alberto Hernando-Calvo, Don Dizon, Shai Magidi, Catherine Bresson, Liang Cheng, Jia Liu, Zhihuang Hu, Crystal Hattum, Tobias Meissner, Ricky Grisson, Liu Liu, Arjun Oberoi, Naftali Z. Frankel, Fanny Wunder, Razelle Kurzrock

**Affiliations:** ^1^Worldwide Innovative Network (WIN) Association - WIN Consortium, Chevilly-Larue 94550, France; ^2^Legorreta Cancer Center at Brown University, Providence, RI 02912, USA; ^3^Vall d’Hebron Hospital Campus and Institute of Oncology (VHIO), Barcelona 08035, Spain; ^4^Tufts Medical Center, Boston, MA 02111, USA; ^5^The Kinghorn Cancer Centre, St Vincent’s Hospital, Darlinghurst, NSW 2010, Australia; ^6^Garvan Institute of Medical Research, Darlinghurst, NSW 2010, Australia; ^7^School of Medicine and Health, University of New South Wales, Sydney, NSW 2052, Australia; ^8^Fudan University Shanghai Cancer Center, Shanghai 200032, China; ^9^Avera Cancer Institute, Sioux Falls, SD 57105, USA; ^10^Chaim Medical Resource, Monroe, NY 10950, USA; ^11^Medical College of Wisconsin, Milwaukee, WI 53226, USA; ^*^These authors contributed equally to this work

**Keywords:** cancer, precision oncology, molecular tumor board

## Abstract

Multiple primary cancers (MPC), the occurrence of two or more distinct malignancies in the same patient, pose significant clinical challenges due to their complexity and the need for individualized treatment strategies. This case, discussed at the WIN Consortium International Molecular Tumor Board, illustrates the clinical journey of a 48-year-old female with a *BRCA1* germline mutation who developed multiple primary cancers: breast, skin, high-grade serous carcinoma of the ovary, colon and small bowel cancers. Her treatment history included doxorubicin and cyclophosphamide for breast cancer, Mohs surgery for basal cell carcinoma, and carboplatin and paclitaxel for ovarian cancer. After participating in the PRIMA trial with niraparib, she was diagnosed with *PIK3CA*-mutated colon cancer, leading to resection and CAPOX chemotherapy. A subsequent diagnosis of small bowel adenocarcinoma, molecularly resembling her colon cancer, along with a unique BRCA1-STARD3 fusion in inguinal lymph nodes prompted a comprehensive MTB review. The panel discussed several precision strategies, including combinations of cetuximab, niraparib, and temsirolimus; FOLFIRI with anti-EGFR inhibitors; anti-CTLA-4 and anti-PD-1 (botensilimab and balstilimab); regorafenib with anti-CTLA-4 and anti-PD-1 (ipilimumab and nivolumab); trifluridine/tipiracil with bevacizumab; regorafenib alone; aspirin for potential benefits in patients with *PIK3CA* mutations; therapies targeting *PIK3CA* and *EGFR* alterations; regular imaging and liquid biopsies assessments for monitoring disease progression and guide future treatment decisions; and vaccines targeting PIK3CA, STARD3 and TP53. This case underscores the complexity of managing MPC and highlights the essential role of international molecular tumor boards in generating innovative, tailored treatment recommendations for patients with rare and multifaceted disease courses.

## INTRODUCTION

Patients who develop multiple cancers, often referred to as “multiple primary cancers” (MPC), represent a unique and challenging subset in oncology. MPC, defined as the occurrence of two or more distinct malignancies in the same patient, accounts for an estimated 2–17% of all cancers [1, 2]. Although relatively rare, MPC pose significant challenges in terms of diagnosis, treatment, and management. Patients diagnosed with one primary cancer have an elevated risk of developing additional primary malignancies, either synchronously (occurring within a short, predefined interval of the first primary tumor, commonly 2 months per SEER-criteria or 6 months per common international convention) or metachronously (occurring after that interval) [3, 4]. This phenomenon can arise from several factors, including genetic predispositions, environmental exposures, previous cancer treatments, and lifestyle factors [5, 6]. Inherited genetic mutations, such as those in the *BRCA1/BRCA2* genes, significantly increase the risk of developing multiple types of cancers by impairing DNA repair mechanisms of the homologous recombination repair (HRR) pathway, leading to a higher likelihood of new, independent malignancies over time [2, 7–9]. Additionally, patients treated for one cancer, particularly with radiation or certain chemotherapies such as alkylating agents, platinum-based drugs and anthracycline topoisomerase II inhibitors, may have an elevated risk of developing a second malignancy due to induced DNA damage resulting in new cancer years later [10–13]. Continued exposure to carcinogens, such as tobacco smoke or certain occupational hazards, can also result in new primary cancers [14, 15]. Also, lifestyle factors such as diet, physical activity, and alcohol consumption may contribute to cancer development [16]. The presence of multiple malignancies complicates treatment, as therapies effective for one cancer might not be suitable for another, particularly if the cancers have differing molecular profiles [17]. This requires personalized treatment plans and close monitoring. Studies have shown that cancers in patients with MPC often present with unique molecular characteristics, and certain genetic alterations in a primary cancer may predispose patients to specific types of secondary cancers [1, 18–20]. Understanding and managing MPC is an evolving area of research, with ongoing studies aimed at personalizing treatment approaches and improving long-term outcomes for these patients, including immunotherapies and emerging strategies such as therapeutic cancer vaccines that target patient-specific tumor mutations [21–23].

We present here a case report discussed at the WIN Consortium’s International Molecular Tumor Board (MTB) with experts in precision oncology across the world (Figure 1). This report builds on prior published WIN Consortium’s International MTB case reporting [24]. This case report presents the clinical journey of a 48-year-old female with a *BRCA1* germline mutation who was initially diagnosed with breast cancer and subsequently developed skin, high grade serous carcinoma of the ovary, colon and small bowel cancers. We detail the patient’s relevant medical history, diagnostic workup and the various treatment approaches undertaken, including chemotherapy, targeted therapies and surgeries.

**Figure 1:**
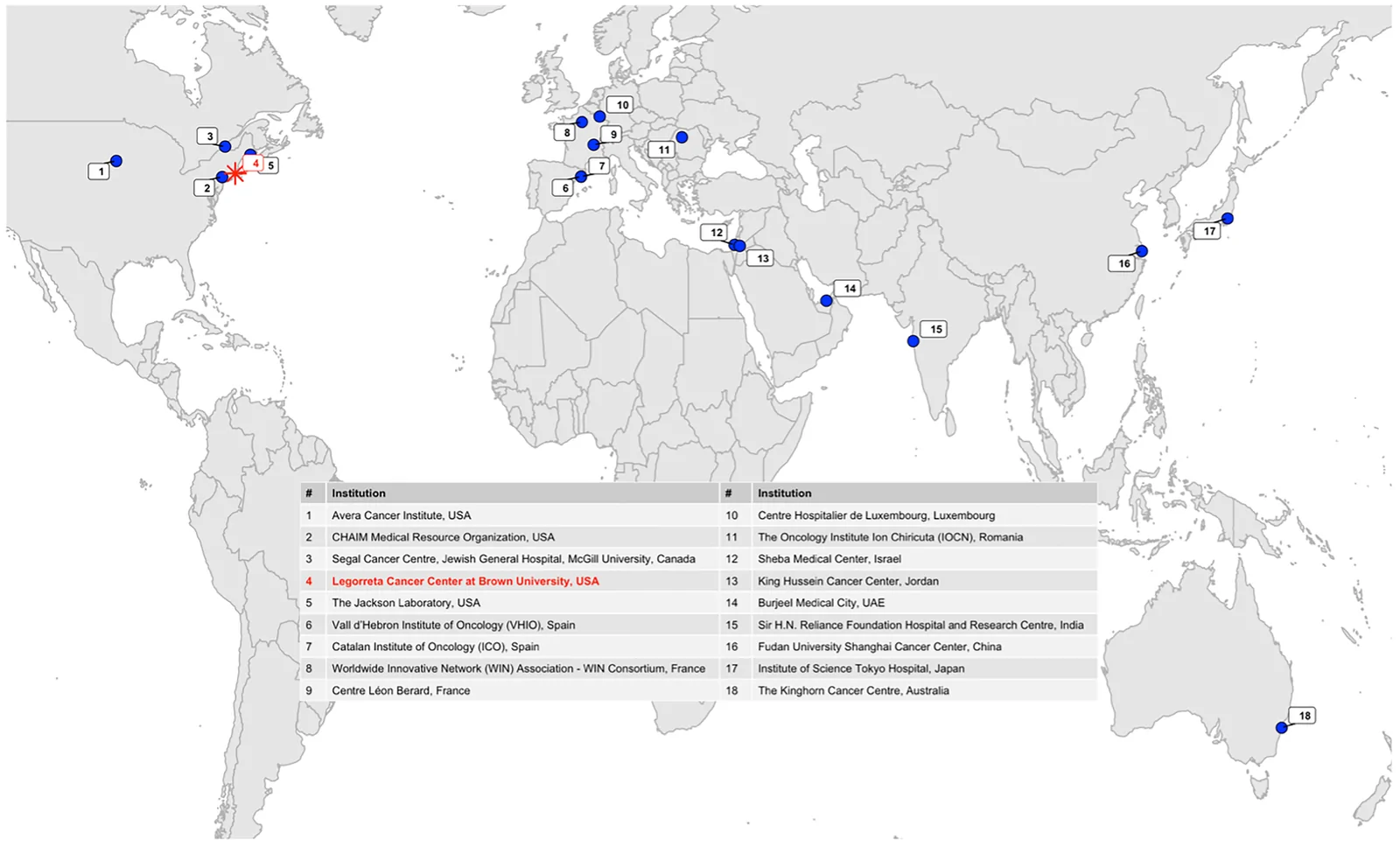
WIN consortium international molecular tumor board participants. The figure highlights 18 institutions participating from 13 countries worldwide (blue dots), with the patient discussed in the MTB originating from USA (red asterisk).

The international WIN MTB panel reviewed the patient’s medical history and genetic profile, discussing a range of treatment options including personalized combination therapies and targeted therapies. The aim was to explore treatment strategies that are specifically tailored to the patient’s distinct genetic and molecular characteristics.

## CASE REPORT DESCRIPTION

The patient is a 48-year-old woman who is a non-smoker and non-drinker with no history of drug use. She carries a *BRCA1 Q1756fs* germline mutation, a well-established founder mutation that is consistent with her Ashkenazi Jewish ancestry. Her family history includes breast, colon and gastric cancers, but no known cases of ovarian cancer. As illustrated in Figure 2, her medical history begins in 2006, at the age of 29, when she was diagnosed with breast cancer. She was treated with doxorubicin hydrochloride (Adriamycin) and cyclophosphamide as neoadjuvant treatment, followed by a bilateral mastectomy. In 2007, she was diagnosed with basal cell carcinoma (BCC) skin cancer located in the nose, that was removed by Mohs surgery.

**Figure 2:**
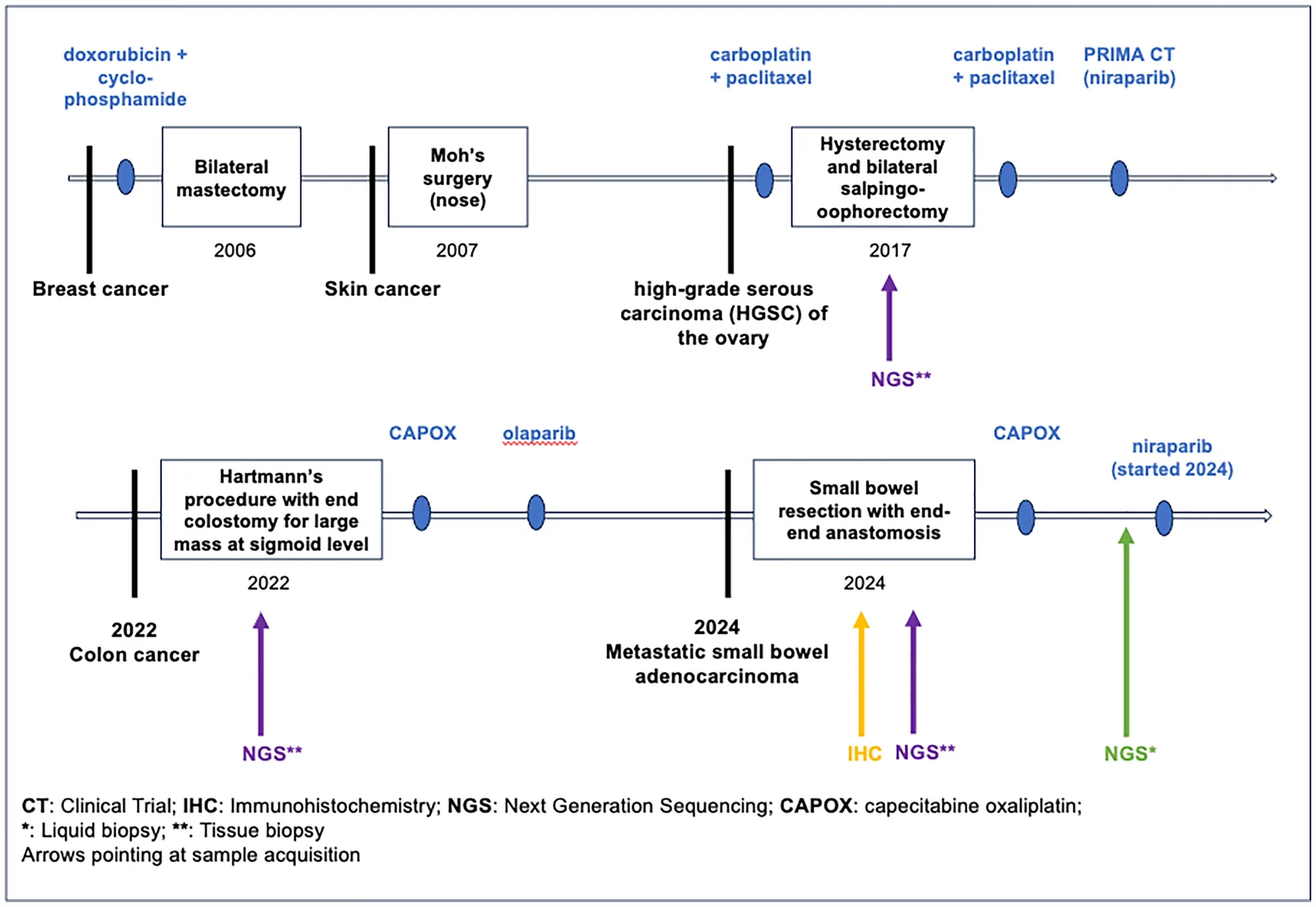
Timeline of treatments and molecular tests. The figure outlines the treatments and molecular tests the patient has undergone throughout her cancer history.

In 2017, she was diagnosed with stage IIIC high-grade serous carcinoma (HGSC) of the left ovary, with metastases in the omentum, associated with a *BRCA1* fusion. She received neoadjuvant and adjuvant treatments with carboplatin and paclitaxel and underwent a prophylactic total abdominal hysterectomy and bilateral salpingo-oophorectomy (TAHBSO) surgery. During the neoadjuvant therapy, she experienced an infusion reaction to paclitaxel; in the adjuvant phase, the paclitaxel was administered every three weeks. It is unclear why paclitaxel was better tolerated in the adjuvant than in the neoadjuvant setting. At the end of 2017, she enrolled in the PRIMA clinical trial for HGSC (NCT02655016), receiving adjuvant niraparib, a PARP inhibitor. By the end of 2018, CT imaging confirmed a complete remission.

In 2022, she presented with minimal rectal bleeding and weight loss, leading to the diagnosis of a large obstructive sigmoid colon mass (stage T3N0), which was later resected. The tumor was microsatellite stable (MSS), and molecular profiling (Table 1) identified a *PIK3CA C420R* mutation with wild-type (WT) *KRAS, NRAS*, and *BRAF.* Immunohistochemistry (IHC) markers for DNA repair proteins involved in the mismatch repair (MMR) system -(MLH1, PMS2, MSH2 and MSH6)– was positive (retained expression), consistent with MMR proficiency by IHC (pMMR). MMR gene sequencing was not performed. Niraparib, which had been taken since 2018, was discontinued in 2022, and she underwent Hartmann’s procedure with an end colostomy for the large sigmoid mass, followed by four cycles of CAPOX (capecitabine and oxaliplatin).

**Table 1:** Molecular alterations

Date of testing		2017	2022	2024			
**Sample**		Tissue (ovary)	Tissue (colon)	Tissue (small bowel)		Tissue (Right inguinal lymph node)	Blood
**Laboratory**		BostonGene	On-site	BostonGene	On-site	BostonGene	Caris
**TMB (mutations/mb)**		1.83 (Low)		4.46 (Low)		2.09 (Low)	Indeterminate
**HRD**		positive				positive	
**Mutations (MAF)**	*PIK3CA* C420R		20.8%	20% GOF			
	*APC* R216^*^			20.5% LOF			
	*TP53* R273H	50.6% LOF				61.5%	
	*TP53* D208E	3.8% LOF					
	*BRAF*	WT	WT	WT		WT	
	*KRAS*	WT	WT	WT			
	*NRAS*	WT	WT				
	*NTRK1/2/3*	WT		WT		WT	
	*RET*	WT		WT		WT	
**Germline testing (MAF)**	*BRCA1* Q1756fs	+		+		+	+ 49.2%
**Amplifications**	*EGFR*			+ >9			
	*TROP2*					+	
**Fusions**	*BRCA1*	+				+ STARD3-BRCA1	
**IHC tests**		HER2 2+ PR negative ER positive	MLH1, PMS2, MSH2 and MSH6 positive		Left parametrium, para rectum: PAX-8, ER and WT-1 positive, CDX-2 negative. Pre sacral tissue: CDX2 positive, CK7 and PAX-8 negative. Right groin lymph node: PAX-8, ER, WT-1, P16 and P53 positive, CDX-2 negative.	ER, PAX-8, WT-1, P16 and P53 positive PGR, GATA-3, mammaglobin, CK20, and CDX-2 negative HER2/neu equivocal (score 2+) MLH1, PMS2, MSH2 and MSH6 positive	
**LOH (%)**	HLA Class I			+			
**Others**	TME	Immune-enriched Fibrotic High PDL1 expression High VEGFR1 expression		No significant findings		Immune-enriched Fibrotic High PDL1 expression High VEGFR1 expression High number of endothelial cells	

Data were provided when available. Only pathogenic alterations are included, and variants of unknown significance (VUS) are not included. Abbreviations: ER: estrogen receptor; GOF: gain of function; HGSC: high-grade serous carcinoma; HRD: homologous recombination deficiency; IHC: immunohistochemistry; LOF: loss of function; LOH: loss of heterozygosity; MAF: mutant allele fraction; PGR: progesterone receptor; TMB: tumor mutational burden; ND: not detected; TME: tumor microenvironment; WT: wild-type.

In early 2023, she began treatment with olaparib (the rationale for administering olaparib rather than resuming niraparib after CAPOX was not documented). A CT scan of the thorax, abdomen and pelvis showed a subtle 12 mm nodular hypodensity in the liver, along with an adjacent 8 mm hypodensity. Further evaluation with MRI characterized the 8 mm lesion as a benign hemangioma, while the 12 mm lesion remained indeterminate, possibly representing a transient attenuation difference (Table 2). Later that year, the patient underwent reversal ileostomy.

**Table 2:** Imaging findings and interpretations

Date	Imaging technique	Site	Findings
January 2023	CT	Thorax, abdomen and pelvis	In the lower portion of liver segment 6, there is a subtle subcapsular nodular hypodensity measuring 12 mm in diameter. Adjacent to this, a smaller 8 mm hypodense area is observed. Additionally, a few scattered tiny millimetric hypodensities are present throughout the liver, though they are too small for detailed characterization.
February 2023	MRI	Abdomen	The prior CT scan identified two areas of hypodensity in segments 6 and 7 of the liver. The area in segment 6 remains indeterminate and may represent a transient attenuation difference. However, the lesion in segment 7 appears more clearly as a benign hemangioma.
January 2024	MRI	Abdomen	No suspicious liver lesion.
January 2024	Unenhanced MRI	Breast	Bilateral retroglandular silicone implants are present. On the left side, there is evidence of an intracapsular rupture, while the right side shows no complications. Findings suggest an intracapsular rupture of the left implant.
June 2024	CT	Thorax, abdomen and pelvis (C+)	There are no pulmonary metastases noted. Interval resection of the small bowel lesion in the right lower quadrant has been performed, with unremarkable findings at the small bowel and colonic anastomosis sites. A soft tissue density in the left hemipelvis is observed, blending with the medial left piriformis muscle. This appearance is unusual for post-surgical changes and may represent interval growth of the previously described speculated lesion, warranting further characterization with a PET scan.
August 2024	PET CT		Physiological head and neck activity is observed with no hypermetabolic cervical adenopathy. There are no FDG-avid lesions in the lungs, liver, spleen, or adrenal glands. No abnormal activity is associated with the breast prostheses. Findings in the left posterior pelvis are most likely related to a postoperative collection rather than recurrent or residual disease, though this cannot be entirely excluded and requires follow-up. The hypodensity seen on CT shows mild uptake with an SUV of 4.6, while the previously noted soft tissue nodule exhibits intense uptake, with an SUV of 14.

In early 2024, a follow-up abdominal MRI showed no suspicious liver lesions. However, a subsequent CT scan of the abdomen revealed a new irregular 1.2 cm nodule at the level of the rectosigmoid anastomosis, suspicious for local recurrence or nodal metastasis, along with a newly enlarged right inguinal lymph node, which was considered a non-specific finding (Table 2).

Later in 2024, following a small bowel obstruction (SBO), she underwent small bowel resection with end-end anastomosis. Pathology confirmed small bowel adenocarcinoma, staged pT4pN0pM1, indicating tumor perforation of the visceral peritoneum, absence of regional lymph node metastasis, and presence of distant metastases. The resected small bowel tumor was microsatellite stable (MSS) and harbored APC R216* mutation, *PIK3CA C420R* mutation (matching the prior colon tumor) and *EGFR* amplification (>9 copies). Additionally, the *BRCA1* germline variant was present, though no rare alterations were identified (Table 1).

During the resection procedure, multiple tumor sites raised concerns about possible metastases from small intestine, ovarian, or recurrent cancer involving the small bowel:

(i) A right inguinal lymph node showed metastatic carcinoma morphologically and immunohistochemically consistent with the patient’s known HGSC of tubo-ovarian origin. The tumor demonstrated MMR proficiency, with positive expression of MLH1, PMS2, MSH2 and MSH6. Additional IHC markers typically associated with HGSC – ER, PAX-8, WT-1, P16 and P53 - were all positive. In contrast, markers commonly linked to breast, gastrointestinal, and colorectal tumors - GATA-3, mammaglobin, CD20 and CDX-2 - were negative. These findings support the presumed tubo-ovarian origin of the tumor.

(ii) A presacral mass of intestinal type moderately differentiated adenocarcinoma, suspected to originate from patient’s known colon carcinoma or the current small bowel carcinoma. IHC staining showed positivity for CDX-2, supporting a gastrointestinal origin, and negativity for CK7 and PAX-8, arguing against a primary tumor of ovarian or other non-gastrointestinal origin.

(iii) An indeterminate peritoneal mass with immunostaining compatible with the patient’s known HGSC. IHC markers typically seen in HGSC - PAX-8, ER, and WT-1- were all positive, while CDX-2 was negative, making a gastrointestinal origin less likely.

Following the small bowel resection, the patient resumed CAPOX therapy. However, she experienced a hypersensitivity reaction to oxaliplatin during her third cycle, leading to discontinuation of the treatment, although she completed two weeks of capecitabine. Subsequently, she resumed niraparib for her ovarian cancer, a treatment which was still ongoing at the time of the case review by the MTB.

By mid-2024, both the gastrointestinal and ovarian cancers showed evidence of activity. Both the cancers carried a germline *BRCA1* variant as well as *BRCA1* fusion involving the *STARD3* gene which was detected in the right inguinal lymph node. STARD3 plays a role in cholesterol transport and is co-amplified and co-expressed with HER2 in breast cancer [25]. The *BRCA1-STARD3* fusion joins two genes located on chromosome 17, with breakpoints 3.38 Mb apart: *STARD3* at chr17:39,637m231 (+ strand) and *BRCA1* at chr17:43,076,614 (- strand). However, her most recent imaging in 2024 showed no lung disease. Although some soft tissue density was noted, a PET CT scan did not reveal concerning abnormalities (Table 2). Molecularly, both her ovarian and small bowel cancers exhibit a very low tumor mutational burden (TMB) of 1.83 mut/Mb and 4.46 mut/Mb, respectively (Table 1).

At the time of the MTB discussion, the patient had no evidence of active disease since resection of the small bowel and ovarian metastasis. Treatment with niraparib, a preventive PARP inhibitor used for patients with germline *BRCA1* mutations, was still ongoing. A liquid biopsy done later in 2024 using the Caris Assure ctDNA panel showed only her *BRCA1* germline mutation (Table 1).

## DISCUSSION

The MTB discussed the best approach for preventing tumor recurrence and managing potential future relapses in this patient. The experts pointed that although the patient carries a *BRCA1* germline mutation, it is unclear whether her colon cancer is driven by the loss of *BRCA1*, even though *BRCA1* carriers have about a 3-fold increased risk of developing colorectal carcinoma (CRC). However, this association is generally weak, and the clinical significance remains uncertain [26]. The *BRCA1* germline mutation was consistently identified across multiple molecular profiles of the patient’s tumors and involved a *BRCA1* fusion in the ovary and a *BRCA1-STARD3* (STAR-related lipid transfer domain-3) fusion in the right inguinal lymph node. Information regarding the precise BRCA1 fusion in the ovary was not available, making it impossible to confirm whether this fusion was also BRCA1-STARD3, as observed in the right inguinal lymph node tissue. To our knowledge, the fusion of *BRCA1-STARD3* has not been previously reported in the literature, making it a novel finding. However, other STARD3 fusions have been reported in gene fusions in gastric cancer [27], acute myeloid leukemia (AML) [28], and is thought to play a role in other cancers [29]. The fusion of *STARD3* with *BRCA1* could potentially result in the loss of BRCA1 function in the second allele. However, there is no evidence of such loss in the patient. In addition, HRD was positive on two occasions (Table 1), supporting an HR-deficient phenotype and making BRCA1 loss-of-function mechanism biologically plausible, although this does not prove the fusion is causal. Importantly, the splicing at the fusion junction at this patient follows canonical splicing signals, supporting the idea that this is a well-formed, biologically plausible event rather than a sequencing artifact. This may lead to a stable RNA and possibly a functional hybrid protein. The fusion was further supported by 15 split reads and 15 spanning reads, providing evidence from two complementary angles that the event is real.

The identification of a novel *BRCA1-STARD3* fusion is hypothesis-generating and raises the possibility that it could contribute to a BRCA1 loss of function phenotype (e.g., by disrupting BRCA1 transcript structure or expression), although the functional impact of this fusion has not been experimentally validated. With respect to STARD3, the functional consequence of the *BRCA1-STARD3* fusion is unknown and could be loss-of-function or gain-of-function like, depending on exon junction/reading frame and domain retention. Clarifying these possibilities would require functional validation. STARD3 is overexpressed in multiple cancer types including as a biomarker of HER2-positive breast cancer. Cholesterol metabolism is a cancer therapy target and STARD3 inhibitors have been developed to interfere with cholesterol transport that may be a viable cancer therapy approach. The finding of a BRCA1-STARD3 fusion in this patient’s ovarian cancer highlights the importance of molecular testing, clinical samples, and discovery of novel and potentially relevant cancer pathogenic mechanisms in translational bedside-to-bench precision oncology. It is believed by the experts that the small bowel tumor is a recurrence of the patient’s previous colon cancer, based on the molecular profiling similarities between the two tumors, which share identical mutation in *PIK3CA (C420R)*. This strongly suggests that the small bowel cancer is derived from the colon cancer, even though such recurrences are unusual. Molecular profiling plays here a critical role in distinguishing whether the tumors represented synchronous primary cancers or metastatic spread. In complex cases, such as MPCs, histopathology alone may be insufficient, whereas next-generation sequencing (NGS) enables comparison of somatic alterations across tumors. The shared *PIK3CA* mutation supports a clonal relationship between the colon and small bowel tumors. In contrast, the ovarian tumor harbors a *TP53* mutation with a very high allele fraction (50.6%), which is absent in the colon tumor, arguing against any germline alteration of *TP53* contributing to the colon cancer. Additionally, the ovarian tumor microenvironment displays high expression of PD-L1, which is not generally observed in colon cancer [30]. This further supports that the patient’s tumors are molecularly distinct.

While the patient is currently in remission and receiving niraparib, the panel of experts discussed potential therapies in case of relapse. Given the targetable alterations present in the patient’s profile, several therapeutic options were proposed including customized combinations of off-label drugs [31–35] based on the I-PREDICT trial approach:

1. Combination of cetuximab (targeting *EGFR* amplification), niraparib (for the *BRCA1* mutation and HRD) and temsirolimus, everolimus or nab-sirolimus (targeting the *PIK3CA* alteration), with temsirolimus being the preferred choice based on clinical experience showing it is more effective. To minimize toxicity of the customized combination, it was discussed to reduce the dose to half for each drug, monitor the patient with clinic visit weekly at first, complete blood count with differential, platelet and metabolic profile and decide if the doses could be increased or decreased depending on the patient’s tolerance. Reducing the dose in this way does not compromise response.
2. Combination of FOLFIRI with an EGFR inhibitor, such as cetuximab or panitumumab, due to the patient’s *EGFR* amplification and the absence of *KRAS*, *NRAS* and *BRAF* mutations.
3. Immunotherapy combination of botensilimab and balstilimab (BOT/BAL), which target CTLA-4 and PD-1, respectively. This combination has shown efficacy in microsatellite-stable (MSS) patients. However, the panel of experts noted that the presence of *EGFR* amplification might increase the risk of resistance or hyper-progression [36, 37], which must be considered. It is not known if combining immunotherapy with an EGFR inhibitor will mitigate the risk of hyper-progression or resistance.
4. Combination of regorafenib with ipilimumab and nivolumab (RIN), though the same caution regarding EGFR amplification and the risk of resistance or hyper-progression applies. At this point in time, it remains unclear whether combining immunotherapy with an EGFR inhibitor will mitigate the risk of hyper-progression.
5. Combination of trifluridine/tipiracil (Lonsurf) with bevacizumab.
6. Regorafenib as a monotherapy.
7. Aspirin, as the experts highlighted that the *PIK3CA* mutation in her colon cancer, which is associated with resistance to EGFR-targeted therapies, has not yet been addressed in clinical trials combining PIK3CA-targeted therapies with EGFR inhibitors for KRAS-WT tumors. A study suggested that aspirin might improve survival for colorectal patients with *PIK3CA* mutations [38]. More recently, results from the ALASCCA trial showed that low-dose aspirin use in patients with PIKC3A-mutated CRC reduced recurrence risk [39]. The patient also has an *APC* mutation, and it is known that non-steroidal drugs like sulindac can downregulate APC through the WNT pathway. However, the effect of aspirin on the WNT signaling is unclear.
8. Temsirolimus and cetuximab targeting *PIK3CA* and *EGFR* alterations, respectively.
9. Bevacizumab, as the patient has a *TP53* mutation, which can upregulate the VEGF/VEGFR pathway and potentially sensitize the tumor to VEGF/VEGFR inhibitors (such as bevacizumab) [35].
10. The use of circulating tumor DNA (ctDNA) monitoring alongside radiological surveillance was discussed as a method for longitudinal tracking of the disease status. Currently available Minimal Residual Disease (MRD) strategies include tumor-informed personalized ctDNA assays (e.g., Signatera) and plasma-only assays incorporating genomic and methylation signals (e.g., Guardant Reveal). In this patient, tumor-informed MRD could enable parallel monitoring by designing separate tumor-specific assays from the colon and ovarian tumors, helping attribute a positive ctDNA signal to the most likely tumor of origin, thereby facilitating precision-based molecular monitoring during surveillance. However, it was emphasized that negative results would not necessarily rule out rare dormant or even active cancer cells below the assay’s limit of detection. Therefore, serial longitudinal testing is more informative than a single post-operative draw. If progression is detected, surgical resection could be considered, followed by molecular profiling to complement pathological assessments and determine whether the recurrence is from ovarian cancer or colorectal cancer. The MRD approach is particularly intriguing, given the patient’s multiple tissue involvement. Unfortunately, a liquid biopsy was only conducted post-operatively, not during active disease, making it challenging to evaluate the significance of negative results.
11. The possibility of using a vaccine targeting PIK3CA and STARD3 mutations, and mutant TP53, was also raised as a potential avenue to explore for the patient.

Further discussions were held between the patient’s clinical team and the MTB experts after the WIN MTB meeting. The option to switch the current therapy (niraparib) to a combination of carboplatin, doxorubicin, and bevacizumab, followed by bevacizumab maintenance until progression or side effects was discussed. This well-tolerated regimen aligns with standard treatment for ovarian cancer rather than colon cancer. At this time, experts agree that adjuvant therapy for colon cancer is not indicated. Alternatively, the option of observing and treating the first recurrence was discussed, though the patient showed less interest in this strategy. The experts considered whether the highest risk of relapse and mortality lies with the colon cancer, which would necessitate prioritizing treatment accordingly.

There was also a discussion about continuing niraparib. The continuation of the same PARP inhibitor is not recommended; indeed, if the patient relapses while on a PARP inhibitor, this may pose risks as shown in the SOLO-3 trial [40]. In addition, PARP inhibitor use carries hematologic toxicity risks including myelodysplastic syndromes (MDS) and acute myeloid leukemia (AML). In a safety meta-analysis of 18 placebo-controlled randomized trials (*n* = 7,307), PARP inhibitors were associated with an increased risk of MDS/AML versus placebo [41]. These events are typically delayed, underscoring the need for long-term vigilance. Importantly, therapy-related MDS/AML risk may be influenced by cumulative prior DNA-damaging exposure. In real-world pharmacovigilance cases with available treatment history, prior therapy consisted mainly of platinum- and taxane-based chemotherapy. Given that the patient was treated with platinum-based chemotherapy and received long-term niraparib, the risk for therapy-related MDS/AML must be considered by incorporating a proactive safety strategy focused on monitoring and early detection of emerging therapy-related malignancies. On the other hand, emerging data suggest there may be benefits to continuing the drug beyond progression, provided additional biomarker-driven therapies are introduced to target co-occurring alterations [42, 43]. Simply continuing the original PARP inhibitor alone is not effective, as the tumor may have developed resistance pathways. Nonetheless, if these resistance pathways are targeted alongside the original treatment, it may yield better results. As the patient was confirmed to be HRD-positive, this supports potential sensitivity to PARP inhibitors and helps rule out the presence of BRCA revertant or hypomorphic alleles that could restore homologous recombination function. Notable, there was no confirmed disease progression on PARP inhibitors. PARP inhibitors were stopped after prolonged use in the metastatic setting. Finally, although fusions are critical drivers and can also yield neoantigens for novel anti-cancer vaccines, the role of the STARD3-BRCA1 fusion in this patient is not known [44].

A platinum-based regimen was discussed if the patient chooses chemotherapy. Platinum also targets *BRCA* altered tumors as it causes DNA damage repair and synthetic lethality. However, if the patient is more than three months post-surgery, the benefit of adjuvant treatment for platinum-sensitive relapse becomes questionable. In this case, monitoring for recurrence and offering chemotherapy later, if ovarian cancer recurrence is confirmed, might be a valid approach. Paclitaxel, doxorubicin, and gemcitabine are all possible options, with bevacizumab as an adjunct. While bevacizumab has shown no benefit in the adjuvant setting for gastrointestinal cancers and is no longer used in maintenance for those cancers, it may still offer benefits in treating ovarian cancer [42, 43, 45–48].

The truncating BRCA1 frameshift (Q1756fs) indicated in this patient is widely classified as pathogenic and is expected to result in loss of BRCA1 function, with disruption of the C-terminal BRCT region that is important for DNA damage response signaling [49–51]. BRCA1 pathogenic variants are heterogenous – some BRCA1 hypomorphic variants (e.g., RING-domain-deficient forms) can retain partial homologous recombination activity and have been associated with relative platinum/PARP inhibitor resistance in experimental systems and recurrent tumors, underscoring the BRCA1 mutation domain can influence phenotype [52]. Consistent with this, studies show that cancer risks vary by BRCA1 mutation location, and emerging clinical datasets suggest that PARP inhibitor benefit may differ by BRCA1 mutation’s type and location, although these were not specifically evaluated in this case [53–55].

This case exemplifies the critical importance of clinical persistence and the transformative potential of precision medicine in complex oncology cases. Comprehensive molecular profiling revealed actionable targets that opened previously unconsidered therapeutic avenues. The identification of alterations provided a molecular roadmap for multiple targeted combination strategies. Clinicians should recognize that initial treatment recommendations—particularly surveillance—warrant periodic reassessment as molecular technologies evolve and our understanding of targetable pathways expands. The availability of off-label combinations based on molecular matching demonstrates that even heavily pre-treated patients with complex histories may benefit from innovative therapeutic approaches. This case highlights that precision medicine is not merely about identifying mutations but about translating molecular insights into clinical opportunities that can extend meaningful treatment options for patients who might otherwise be considered to have exhausted conventional therapies. For clinicians, the lesson is clear: molecular profiling should be viewed as an evolving clinical tool that can reveal new therapeutic possibilities, and patient care decisions should remain dynamic rather than definitive, particularly in the era of rapidly advancing precision oncology.

A limitation is the lack of longitudinal serum tumor marker measurements. Nevertheless, in the context of multiple primary malignancies, such tumor markers may have limited interpretability because elevated levels may lack specificity for the tumor of origin and may, therefore, provide limited clinical clarity.

In conclusion, the discussions prioritized carboplatin and bevacizumab, followed by maintenance therapy with niraparib, bevacizumab, and aspirin, along with a potential vaccine targeting PIK3CA, STARD3, and mutant TP53. A PARP inhibitor can be re-introduced if platinum needs to be discontinued. Regular follow-up with imaging every three months and ctDNA monitoring every 4–6 weeks was also advised, at least for the coming year.
